# Can antioxidant treatment replace delay in bracket bonding? An in vitro study

**DOI:** 10.1186/s12903-023-02894-3

**Published:** 2023-04-02

**Authors:** Shaimaa S. Zaki, Sayed M. Ghorab, Marwa A. Tawfik, Marwa S. Shamaa

**Affiliations:** 1 Department of Orthodontics, Faculty of Dentistry, Mansoura University, Mansoura, 35516 Mansoura Egypt; 2 Department of Dental Biomaterials, Faculty of Dentistry, Mansoura University, Mansoura, Egypt

**Keywords:** Antioxidants, Alpha-tocopherol, Green tea, Sodium ascorbate, Bleaching, Shear bond strength

## Abstract

**Background:**

Deterioration in shear bond strength has been reported after immediate bracket bonding following hydrogen peroxide bleaching. This study compared the effectiveness of three antioxidant agents, namely, alpha-tocopherol, green tea extract, and sodium ascorbate, in reversing the bleaching effect and as possible alternatives to delayed bonding.

**Methods:**

A total of 105 extracted human premolars were arbitrarily assigned to 7 groups (*n* = 15 each), including group 1 as the unbleached control group and six experimental groups, which were bleached with 40% hydrogen peroxide in three sessions of 15 min each. In experimental group 2, bonding was performed immediately after bleaching, whereas in groups 3 and 4, bonding was delayed for 1 and 2 weeks, respectively; meanwhile, the specimens were immersed in artificial saliva at 37 °C. Groups 5, 6, and 7 were treated immediately after bleaching with 10% of alpha-tocopherol, green tea extract, and sodium ascorbate solutions, respectively, for 15 min. Specimens were processed using 500 thermal cycles between 5 and 55 °C, with a dwell time of 30 s after 24 h of bracket bonding, and then tested for shear bond strength. The adhesive remnant index was examined to evaluate fracture mode. One-way analysis of variance, Kruskal–Wallis *H*, and *post hoc* Tukey’s honestly significant difference tests were used to compare the data. Significant results were subjected to pairwise comparisons with Bonferroni’s correction-adjusted of *p* values ≤ 0.050.

**Results:**

Shear bond strength was significantly lower (*p* < 0.001) in the immediate bonding and 1-week delay groups than in the control group. However, no significant difference was detected among the 2-week delay, antioxidant-treated, and control groups (*p* > 0.05).

**Conclusions:**

Application of 10% alpha-tocopherol, green tea extract, or sodium ascorbate for 15 min could restore shear bond strength after 40% hydrogen peroxide bleaching as an alternative to delay in bracket bonding.

## Background

In 1884, Harlan [1] introduced the concept of teeth bleaching using hydrogen peroxide (HP). Since then, considering the growing demand for aesthetic procedures, HP bleaching has been used as a minimally invasive approach for in-office management of tooth discoloration with immediately visible results.

HP bleaching releases free radicals of hydroxyl, per-hydroxyl, peroxide, singlet oxygen, and superoxide anions that target the double bond of the chromogenic complex structures causing tooth discoloration, thereby eventually reducing the discoloration over time [2, 3]. Immediate amelioration in tooth color could raise the patients’ ambitions of having their teeth aligned. However, deterioration in the shear bond strength (SBS) of orthodontic brackets after the application of HP was reported because the residuals of free radicals in tooth structures hinder complete resin polymerization by binding to the polymer chains in adhesive resin, Consequently, the elongation of the polymer chain is terminated and the SBS of brackets to tooth structures decreases [4].

Fortunately, the reduction in SBS appears to be transient, and a recovery period after bleaching, ranging from 24 h to 2 weeks or more, has been recommended [5–8]. Some strategies for avoiding delays in bonding involve treating enamel with alcohol, adhesives with organic solvents, or removing the superficial enamel layer [9–11]. Moreover, the application of different antioxidant agents after HP bleaching instantaneously boosts SBS. These antioxidants act as scavengers of residual free radicals by donating their free electrons, thereby ending the electron-taking reaction of the bleaching process and promoting complete resin polymerization. Several researchers have hypothesized that the immediate application of antioxidants, including ascorbic acid, sodium ascorbate (SA), proanthocyanidins, grape seed, cranberry, extracts of pine bark, alpha-tocopherol (α-T), sodium bicarbonate, aloe vera, and green tea (GT) extracts, would reverse the reduction in SBS [10, 12–15].

α-T, an active component of the vitamin E complex, is the most effective lipid-soluble antioxidant in nature [15, 16]. GT, which comprises flavanols or catechins that help scavenge excess free radicals, has been used as a promising antioxidant [16, 17]. Ascorbic acid (vitamin C) is believed to improve bond strength; however, the low pH of ascorbic acid (approximately 4.0) can lead to over-conditioning of the enamel surface. Therefore, SA, the neutral salt of ascorbic acid (pH of approximately 7.0), is more applicable intraorally [14, 18, 19].

This study aimed to compare the in vitro effectiveness of the immediate application of three antioxidant agents (10% α-T, 10% GT, and 10% SA) on SBS and investigate whether such treatment could be considered an alternative to delayed bonding of orthodontic brackets after 40% HP bleaching.

## Methods

The ethical committee of Mansoura University Dental Faculty, Mansoura, Egypt, provided institutional approval for this study (#A08030821).

### Specimen preparation

A total of 105 human premolars extracted for orthodontic treatment were collected. Teeth had a sound enamel buccal surface; were devoid of caries, cracks, fracture, fluorosis, restoration, or any developmental anomalies; and had not been treated previously with any chemicals. Debris was removed from the collected teeth using a manual periodontal curette. Then, the teeth were polished using a low-speed handpiece (APPLEDENTAL, Guangdong JINME Medical Technology Co. Ltd., Foshan, China) with a coolant system and oil-free, fluoride-free polishing paste (PROPHY PASTE; PSP Dental Co. Ltd., Kent, UK). Subsequently, the teeth were preserved at room temperature in distilled water, which was changed daily, until usage. Thereafter, each tooth was adjusted within a 15 X 15-mm self-curing acrylic resin block (Acrostone, Cairo, Egypt), embedding the root up to the cemento–enamel junction in which each tooth was manually placed in a central position so that the buccal surface was set perpendicular to the block base.

**Fig. 1:**
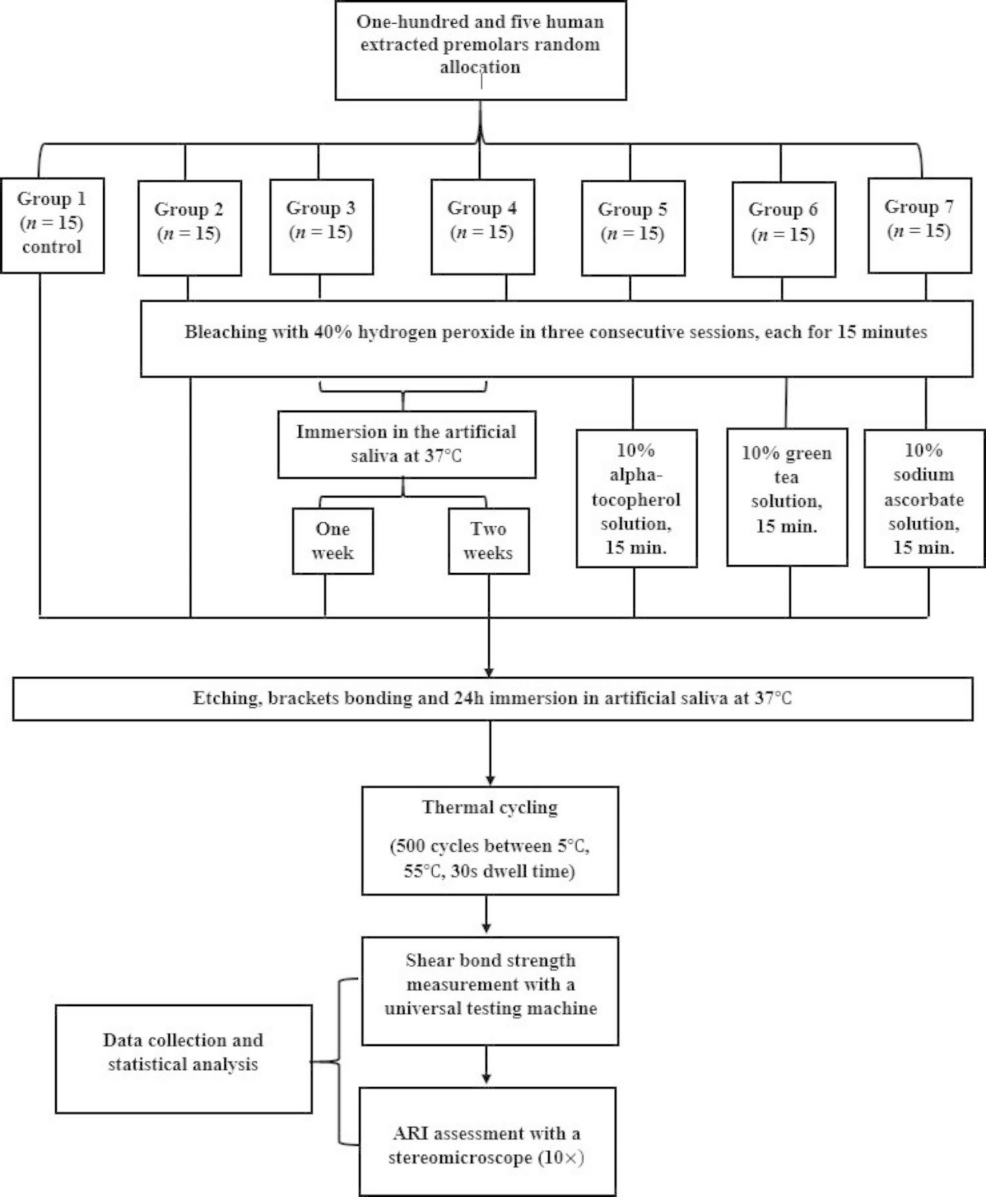
Flow chart of treatment procedures and methodology in each study group

The specimens were arbitrarily assigned to seven groups (*n* = 15 for each; Fig. 1), including a control group and six experimental groups. Specimens in group 1 (control group) were not subjected to bleaching or antioxidant application. They were immediately bonded to brackets and preserved at 37 °C in artificial saliva, which was prepared using 19 mg/L MgCl_2_.6H_2_O, 544 mg/L KH_2_PO_4_, 2240 mg/L KCl, and 103 mg/L of CaCl_2_ (pH ~ 7.0) at the Analytical Chemistry Department, Faculty of Pharmacy, Mansoura University, Mansoura, Egypt [20]. Specimens in all experimental groups (groups 2–7) were subjected to 40% HP bleaching. After bleaching, teeth in group 2 were immediately bonded without the application of any antioxidant; teeth in group 3 were immersed in artificial saliva at 37 °C (changed daily) for 1 week before bonding without the application of any antioxidant; teeth in group 4 were immersed in artificial saliva at 37 °C (changed daily) for 2 weeks before bonding without application of any antioxidant; teeth in group 5 were treated with 10% α-T solution immediately after bleaching before bonding; teeth in group 6 were treated with 10% GT solution immediately after bleaching before bonding; and teeth in group 7 were treated with 10% SA solution immediately after bleaching before bonding.

### Bleaching procedures

The buccal surface of each tooth in all six experimental groups was exposed to 40% chemical-activated HP gel (POWER WHITENING YF; WHITEsmile, GmbH, Birkenau, Germany). According to the manufacturer’s instructions, bleaching was performed in three consecutive sessions, each for 15 min. A 1–2-mm-thick layer was applied with continuous stirring every 5 min with a pointed sterile probe countervailing the evaporated agent. The bleaching gel was then aspirated with high-power dry suction after each session, and the specimens were rinsed with water after the last session and dried.

### Preparation of antioxidant solutions

To prepare 10% α-T solution, 23 mL of α-T retrieved from capsules (Spondex; Atos Pharma, Cairo, Egypt) was dissolved in 230 mL of ethanol. To prepare 10% GT solution, 23 g of GT retrieved from grinding pills (Nature’s Garden Green Tea Extract; Nature’s Garden, Holland and Barrett, Nuneaton, UK) was dissolved in 230 mL of distilled water. To prepare the 10% SA solution, 23 g of SA powder (prepared at the Chemistry Department, Faculty of Agriculture, Mansoura University, Mansoura, Egypt) was dissolved in 230 mL of distilled water. Each solution of antioxidant was kept in a sealed, dark glass sampling bottle and used fresh.

### Application of antioxidants

Each antioxidant solution was applied once on the bleached buccal surface of teeth for 15 min at a continuous rate of 1 mL/minute using a syringe. Specimens treated with GT and SA were then rinsed with distilled water and those treated with α-T were rinsed with ethanol. All specimens were air-dried gently before the bonding procedures.

### Bonding procedures

Etching with 37% phosphoric acid gel (N-Etch; Ivoclar Vivadent Inc., Amherst, NY, USA) was performed for 30 s on the buccal surface of the teeth. Then, the teeth were rinsed with water/air spray for 30 s and dried thoroughly until they had a frosty white appearance. Next, the primer (Transbond™ XT Light Cure; 3 M Unitek, Monrovia, CA, USA) was applied to the enamel surface using a microbrush.

Standard edgewise metallic premolar brackets with a 0.022-inch slot (American Orthodontics, Sheboygan, WI, USA) were attached to the centres of the buccal surfaces of the teeth with the adhesive system (Transbond™ XT Light Cure; 3 M Unitek, Monrovia, CA, USA). Each bracket was loaded onto the enamel surface using a bracket holder and pressed through the bracket slot perpendicularly on the bracket base by the flat end of the tensiometer (75.02.008; Morelli Ortodontia, Sorocaba, Brazil) with a standard force of 300 g. Excess adhesive was removed using the back of a bracket holder. Then, light curing (BlueLEX LD-105, Monitex Industrial Co., Taipei, Taiwan) with an intensity of 1000 mW/cm^2^ verified using a curing radiometer (Bluephase Meter II, Ivoclar Vivadent Inc., Amherst, NY, USA) before each application, was performed for 40 s (20 s on the occlusal surface and 20 s on the gingival surface). All specimens were stored by immersing them in artificial saliva in a sealed container at 37 °C for 24 h until polymerization was complete [21].

### Thermal cycling

After 24 h of bonding, the specimens were subjected to 500 thermal cycles [21] in water baths, with the temperature of 5 °C and 55 °C and a dwell time of 30 s at each temperature and 15 s for air transfer (ROBOTA Industries, Alexandria, Egypt), representing 10–25 clinical days [22].

### SBS test

A Universal testing machine (Model 3345 with Instron Bluehill 3 software; Instron Inc., High Wycombe, UK) was used for the SBS test with a compressive force, which was in the occlusal–gingival direction onto the tooth surface–bracket base interface with a crosshead speed of 1 mm/min until the bracket was detached. The fracture load was determined in newtons and divided by the surface area (10.55 mm^2^) of the bracket base to convert to mega-pascals (MPa).

### Adhesive remnant index

To authenticate the fracture mode of the specimens, the adhesive remaining on the enamel surface was evaluated by two examiners using a stereomicroscope (SZ2-ILST; Olympus, Tokyo, Japan) under 10× magnification. The adhesive remnant index (ARI) was classified as follows [23]:

- 0: No resin remained on the enamel.
- 1: Less than 50% of resin remained on the enamel.
- 2: More than 50% of resin remained on the enamel.
- 3: All resin remained on the enamel.

### Statistical analysis

One-way analysis of variance was performed to compare the SBS values of the study groups after using Shapiro–Wilk’s test to confirm the normality of the data (*p* > 0.050). Significant results were evaluated with *post hoc* Tukey’s honestly significant difference (HSD) test to determine where that difference subsisted. The Kruskal–Wallis *H* test was applied to compare the ARIs of the study groups after using the Kappa test (0.857) to assess inter-examiners’ reliability, which confirmed a non-significant difference. Significant results were subjected to pairwise comparisons with Bonferroni’s correction-adjusted *p* values. The significance level (*p*-value) for applied tests was ≤ 0.050. All data were processed with SPSS Statistics, version 26.0 (IBM Corporation, Armonk, NY, USA).

## Results

The comparison between SBSs of the groups (means ± standard deviations), listed in Table 1, revealed a significant difference among the study groups. Pairwise comparisons of SBSs revealed that the SBSs of experimental groups 2 (immediate bonding) and 3 (1-week delay) differed significantly from that of the control group (*p* < 0.001), whereas the SBS of the control group did not differ significantly from those of groups 4 (2-week delay; *p* = 1.000), 5 (10% α-T treatment; *p* = 0.829), 6 (10% GT treatment; *p* = 0.755), and 7 (10% SA treatment; *p* = 0.695). Furthermore, the SBS of group 4 did not differ significantly from that of group 5 (*p* = 0.964), group 6 (*p* = 0.931), or group 7 (*p* = 0.899), and the SBSs of groups 5, 6, and 7 revealed no significant difference from one another (*p* = 1.000).

**Fig. 2:**
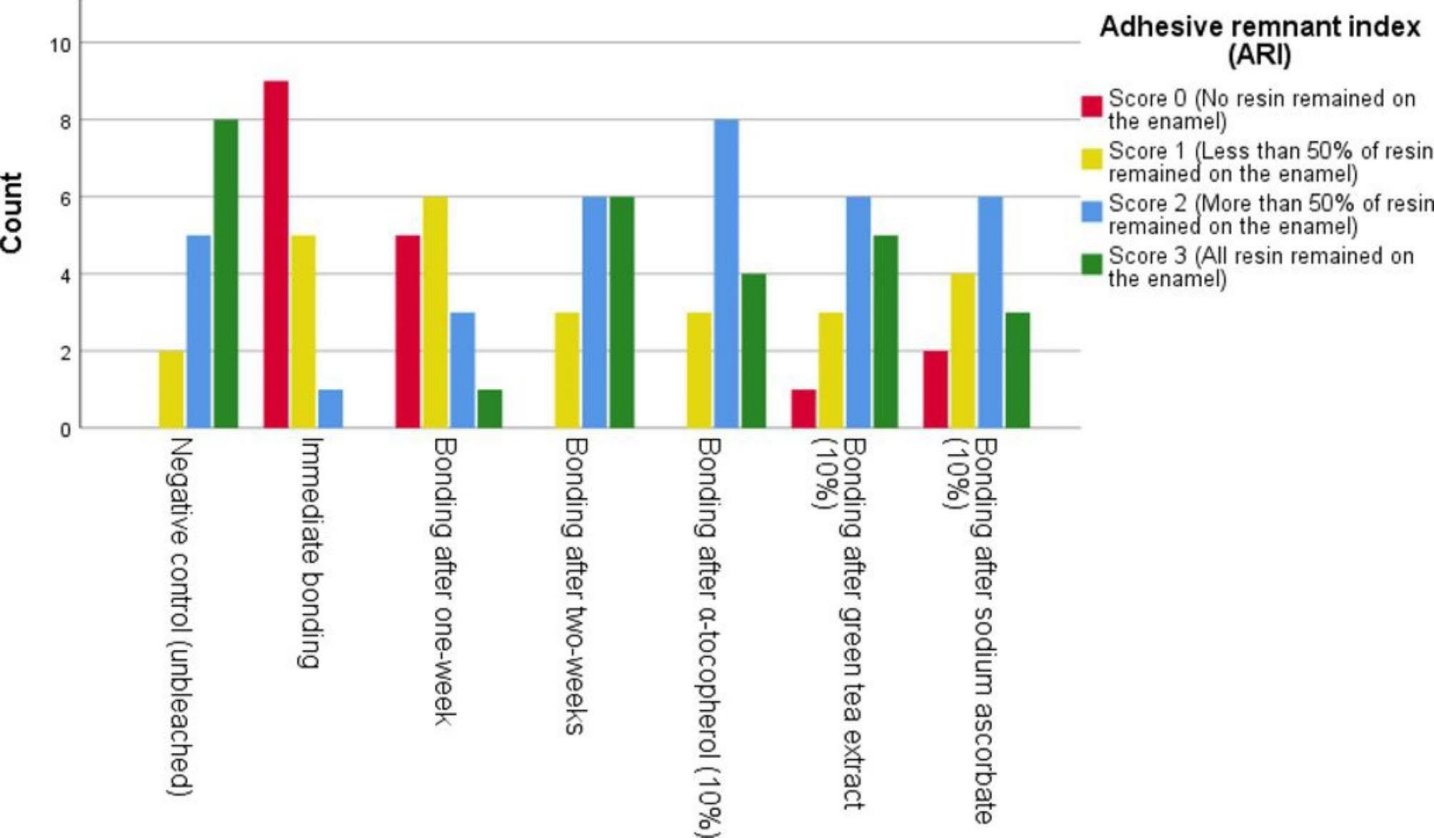
Frequency of ARIs in the study groups *ARI* adhesive remnant index. Test of significance was the Kruskal–Wallis *H* test; *H* = 40.084 with a degree of freedom of 6. Significance level was at *p* ≤ 0.050

The results of stereomicroscopic analysis of the specimens are presented in Fig. 2. The frequencies of ARIs of 0 and 1 were high in groups 2 and 3, which differed significantly from those in the control group (*p* < 0.05). By contrast, the frequencies of ARIs of 3 and 4 were high in groups 5, 6, and 7, with no significant difference from the control group (*p* > 0.05).

**Table 1:** Comparison of shear bond strengths in the study groups

Group	Mean (MPa)	Standard deviation (MPa)
1 (Control)	19.3^a*^	2.8
2	9.4^b*^	3.1
3	11.6^b*^	2.8
4	18.9^a*^	2.6
5	18.0^a*^	2.9
6	17.8^a*^	2.3
7	17.7^a*^	2.5

^*^Different letters indicate a significant difference. *Post hoc* Tukey’s honestly significant difference test with Bonferroni’s correction-adjusted *p* values for pairwise comparisons. A *p*-value of ≤ 0.050 was considered significant

*F* of one-way analysis of variance = 31.273, Partial η2 (0.657) is a measure of the effect size (*f* = 1.383999)

## Discussion

Although tooth bleaching has a great aesthetic value, it has some deleterious effects on tooth structures, such as increased porosity, decreased microhardness, hypersensitivity, and reduced bond strength of orthodontic brackets to tooth structure [2, 24–26]. Vital teeth can be bleached with HP or carbamide peroxide at different concentrations. HP in high concentrations has been reported to have more adverse effects on tooth structures than carbamide peroxide [7, 27].

In the present study, SBS reduced significantly when bonding was performed immediately after 40% HP bleaching. When bleaching was followed by a 1-week delay before bonding, SBS showed some improvement. However, a 1-week delay was not comparable enough with that in the unbleached control group, whereas a 2-week delay seemed to be appropriate for the recovery of SBS after bleaching. According to Dishman et al. [4], SBS is reduced after HP bleaching because the residual free radicals interfere with complete resin polymerization. Perdigão et al. [28] referred to the reduction in SBS as the alteration in organic/inorganic enamel structure. Electron microscopy revealed poor, sparse, and defective resin tags in bleached enamel but not in unbleached enamel [29, 30]. The reduction in SBS was considered temporary because free radicals of HP that substitute hydroxyl ions in apatite crystals eventually dissociate from those crystals [31, 32].

Different modalities have been used to neutralize the residual free radicals of bleaching and determine an alternative to delay in bonding. Results of several studies have indicated that various antioxidants can reverse the reduction in SBS after bleaching [10, 12–15, 33]. α-T represents the most potent form of vitamin E and acts as the first line of cell defense in the human body against free radicals. It is a lipid-soluble carotenoid that is easily soluble in ethanol [10, 15, 16]. The antioxidant properties of GT are attributed to flavonoids, which represent 94% of its phenolic content and include various catechins, such as catechin, epicatechins, and epigallocatechin-3-gallate. GT has been described as a promising agent with proven efficiency against the adverse effects of bleaching [16, 17]. SA is a biocompatible, non-enzymatic, neutral salt of ascorbic acid [19]. Moreover, 10% SA is considered the “gold-standard” of post-bleaching antioxidants. Lai et al. [18] reported its efficacy on dentin bonding strength after bleaching with HP or sodium hypochlorite.

The action of the α-T, GT, and SA solutions have been compared in terms of different factors, including time of application, concentrations, and physical forms to determine the most efficient protocol to be used after HP bleaching. Controversy exists in the literature about the influence of these factors in boosting SBS. Freire et al. [19] emphasized that a 5-min application of SA after HP bleaching was sufficient. In another study, Freire et al. [34] obtained the same result through two 1-min applications of 35% SA after HP bleaching. In some previous studies, a 10-min antioxidant application was considered sufficient for restoring SBS after bleaching [35, 36], whereas in others, a 10-min application of antioxidants after HP bleaching improved SBS but did not help it recover in comparison with the unbleached teeth [10, 37]. Other studies have shown that a 15-min application of antioxidants at a concentration of 10% after HP bleaching restored bonding strength [9, 38, 39]. Antioxidant application for at least 60 min was reported to counteract bleaching effects [40]; however, 60 min is considered too long for clinical application. Another study revealed that prolonged SA application reduced bond strength because acid etching could not eliminate the precipitated remnants of SA crystals [41]. Lai et al. [42] recommended that one-third of bleaching time would be convenient for antioxidant application to reverse the reduction in SBS. Therefore, in this study, a 15-min application of antioxidants seemed adequate after three 15-min bleaching sessions.

Türkün et al. [43] reported that 2.5% and 5% of SA were not sufficient to improve SBS, whereas 10% SA was sufficient. Briso et al. [44] found that 10% SA was effective after carbamide peroxide bleaching but not after HP bleaching, whereas Vidhya et al. [36] revealed that 10% SA was sufficient after HP bleaching. Solution and gel forms of antioxidants were reported to eventually reverse the bleaching effect [45]. However, Sasaki et al. [15] reported that the solution form of α-T was more effective for SBS after bleaching than the gel form. The gel form may be easier to apply clinically but could slow down the rate of antioxidant release and thus prolong the application of antioxidants [43].

Therefore, this study analyzed the antioxidants in standardized 10% concentrations for 15 min in a solution form as the target was to obtain an efficient protocol with both the lowest concentration and the minimum application time to avoid overexposure. The findings of this study revealed that 15-min applications of 10% α-T, 10% GT, and 10% SA restored bond strength after 40% HP bleaching and that the effects of a 2-week delay in bonding did not differ significantly from those of the unbleached control group. These findings were similar to those of Murad et al. [38], although they used SA in the gel form after 37.5% HP bleaching. Thus, further investigations should be conducted for determining the correlation between the time of application of both HP bleaching and antioxidants, regardless of the concentration of HP bleaching or the physical form of the antioxidants.

This study did not reveal any significant difference in SBS among the three antioxidant-treated groups. However, SBS was non-significantly higher with α-T, which may be attributed to the synergistic effect of ethanol used for its dissolution, in concordance with the finding of Sasaki et al. [15], and was non-significantly higher with GT than with SA. Nari-Ratih and Widyastuti [10] concluded that α-T and SA application resulted in non-significantly higher SBS than did GT application and stated that the difference may be attributed to the molecular weights of these agents. Antioxidants were applied for different times in the two studies, which could account for the difference in findings and suggest that longer application of GT is required to achieve its effect. Therefore, further studies are required to explain the non-significant differences among antioxidant-treated groups.

The adequate bond strength of metallic orthodontic brackets has been reported to range from 5.9 to 7.8 MPa for in-vivo and 4.9 MPa for in-vitro performance [46]. Therefore, the recorded SBSs of all groups in this study could be adequate for metallic orthodontic brackets bonding.

To determine the fracture mode of specimens, ARI was evaluated [23] using stereo-microscopy with 10× magnification. An ARI score of 2 (more than 50% of resin remained on enamel) was predominant in experimental groups 4, 5, 6, and 7, with no significant difference in comparison with the control group. In contrast, in groups 2 and 3, ARI scores of 0 (no resin remained on enamel) and 1 (less than 50% resin remained on enamel) predominated, which could be correlated to the reduction in SBS after bleaching.

Therefore, according to these findings, at least a 2-week delay in bonding is a superior protocol for completely restoring SBS after 40% HP bleaching; the discussed protocol involving antioxidants is an effective alternative.

In this study, specimens of human teeth were used, exposed to 500 thermal cycles, and preserved in artificial saliva at 37 °C to simulate a clinical situation. However, it was an in vitro study. Thus, more clinical trials are recommended with a variety of concentrations, application times, and bleaching agents. Furthermore, different antioxidants are recommended to be compared in terms of color analysis to determine how much they alert the bleached shade. The availability of commercial products containing antioxidants with a long shelf-life and stability at room temperature is required.

## Conclusions

- Application of α-T, GT, and SA had a similar efficacy in restoring SBS after 40% HP bleaching.
- Application of α-T, GT, or SA in 10% concentration for 15 min immediately after bleaching provided an alternative to delay in bracket bonding after bleaching.

## Data Availability

The datasets used and/or analyzed during the current study are available from the corresponding author upon reasonable request.

## Abbreviations

HP: Hydrogen peroxide
SBS: Shear bond strength
α-T: Alpha-tocopherol
GT: Green tea extract
SA: Sodium ascorbate
ARI: Adhesive remnant index
Tukey’s HSD: Tukey’s honestly significant difference.
